# Special Issue: “Non-Destructive Testing of Structures”

**DOI:** 10.3390/ma13214996

**Published:** 2020-11-06

**Authors:** Magdalena Rucka

**Affiliations:** Department of Mechanics of Materials and Structures, Faculty of Civil and Environmental Engineering, Gdańsk University of Technology, Narutowicza 11/12, 80-233 Gdańsk, Poland; magdalena.rucka@pg.edu.pl or mrucka@pg.edu.pl; Tel.: +48-58-347-1891

**Keywords:** non-destructive testing, structural health monitoring, civil engineering structures, mechanical structures, damage detection and visualization, modeling and simulations

## Abstract

The Special Issue “Non-Destructive Testing of Structures” has been proposed to present recent developments in the field of diagnostics of structural materials and components in civil and mechanical engineering. The papers highlighted in this editorial concern various aspects of non-invasive diagnostics, including such topics as condition assessments of civil and mechanical structures and connections of structural elements, the inspection of cultural heritage monuments, the testing of structural materials, structural health monitoring systems, the integration of non-destructive testing methods, advanced signal processing for the non-destructive testing of structures (NDT), damage detection and damage imaging, as well as modeling and numerical analyses for supporting structural health monitoring (SHM) systems.

Engineering structures are gradually destroyed over time due to the influence of atmospheric conditions, excessive loads, and processes of natural aging. Since damage in a structural element may lead to improper operation of the whole object, various damage detection and structural health monitoring (SHM) methods have thus been investigated and developed to improve reliability and safety and to solve the maintenance problems of infrastructural and mechanical structures.

The term non-destructive testing (NDT) covers a wide group of measurement and analysis techniques used in the process of assessing the current state of structural materials or elements. It is a quick and effective approach, the main advantage of which is the ability to examine the material in a non-invasive manner, without damaging or changing the composition or shape of the inspected object. In recent years, there has been growing interest in the development of non-invasive methods, including in the field of civil and mechanical engineering.

The Special Issue “Non-Destructive Testing of Structures” has been proposed to gather the experience of civil and mechanical research communities in relation to the latest advances and trends in the field of diagnostics of structures and their components. A total of 22 papers were published in the Special Issue to touch different aspects connected with novel NDT approaches, with particular emphasis on the development of single and integrated measurement techniques, damage imaging procedures, advanced signal processing as well as modeling and numerical analyses for supporting SHM systems. A brief description of the Special Issue papers is presented below.

A wide range of experimental and numerical studies is reported in the papers in this Special Issue [1,2,3,4,5,6,7,8]. Nazarko and Ziemiański [1] monitored the axial bolt forces by means of elastic wave propagation signals. A series of laboratory tests were carried out on flange connections with six bolts, subjected to static tensile tests. Some bolts were equipped with washer load cells for the precise measurement of axial force. Moreover, selected bolts were equipped with piezoelectric transducers (actuator and sensor working in a pitch-catch configuration) in order to register the elastic wave signals. The results of ultrasonic testing were next integrated with the artificial neural network (ANN) for both signal compression and as an interface tool. The results showed that ANNs were able to predict the axial forces in bolts with relatively good accuracy. The proposed approach revealed the significant potential for real-life NDT inspections [1]. The effect of cladding stiffening on steel structures was studied by Korcz-Konkol and Iwicki [2]. Their work was undertaken to analyze what kind of non-destructive approach can be used for the condition assessment of existing buildings. Experimental investigations on small-scale shear panels made of trapezoidal sheeting were conducted to observe the behavior of the diaphragm under increasing and repeated load. The force–displacement relation and strains in selected areas of the sheeting were recorded during tests in a testing machine. Additionally, numerical calculations by the finite element method (FEM) in Abaqus software were conducted. The results obtained revealed the nonlinear, hysteretic force–displacement behavior of the panel and the occurrence of persistent deflections and stresses remaining even after the unloading. The authors concluded that indicators like the stresses, force–displacement paths and modes of failure could be potentially used for structural health monitoring of existing buildings in terms of parasitic stressed-skin action [2]. Palacz et al. [3] presented some aspects of numerical analyses for supporting SHM systems. They concentrated on FEM-based wave propagation modeling. The purpose of the study was to indicate the problems of numerical nature, which are immanent to modelling, as well as practical aspects that may lead to significant errors and the misinterpretation of results. The authors carried out numerical studies on one-dimensional structures. They analyzed the dynamic response covering natural frequencies and modes of natural vibrations and the accuracy of their representations [3]. Li et al. [4] presented a method dedicated to detection, localization, and quantitative analysis of local damage to beam structures based on vibration signal analysis. A novel algorithm based on the unscented Kalman filter was designed. The effectiveness of the method was demonstrated on experimental data acquired for a four-layer frame model composed of plexiglass plates and aluminum columns. The non-destructive testing algorithm was used to evaluate and monitor the strength, stiffness, damping of the frame. The results showed that the proposed damage detection algorithm is much more efficient than conventional methods [4]. Moonens et al. [5] described a promising technology patented under the name effective structural health monitoring (eSHM). In the paper, they addressed the possibility of assessing the fatigue life of a straight lug component by capillary-based structural health monitoring. The eSHM assumes the integrating structurally small and pressurized capillaries into the component. The concept is as follows: a fatigue crack breaches the capillary network and causes a leak to flow to the open atmosphere; next, the loss of pressure in the galleries is detected by a pressure sensor. The concept was verified numerically using the finite element and extended finite element methods. Various capillary sizes and shapes were analyzed. The authors revealed the great potential of the eSHM, especially with the use of additive manufacturing for part production [5]. Liu et al. [6] investigated the influence of a crack size on the stress evaluation of low alloy steel with metal magnetic memory (MMM) technology. A sample made of ferromagnetic material with rectangular grooves of different depths and widths was tested. The metal magnetic memory detector was used to store the *H_p_*(*y*) signal of the sample under different stresses. The fracture morphology was observed by using scanning electron microscopy (SEM). The results showed how different types of grooves influenced the magnetic intensity gradient changes [6]. The paper by Roskosz et al. [7] was focused on the evaluation of the hardness of ferromagnetic steel. Measurements of the Barkhausen noise and hardness were carried out on specimens subjected to plastic strain and thermochemical treatment. A new methodology was developed for the determination of correlations between the Barkhausen noise number of events and hardness [7]. Maciusowicz and Psuj [8] presented a new approach to the non-destructive evaluation of the easy magnetization axis in grain-oriented steel based on the Barkhausen phenomenon and its time–frequency characteristics. In the experiment, a sample of the conventional cold-rolled electrical steel sheet was used. Measurements were made for several angular settings towards the rolling and transverse directions. A procedure of signal transformation into the time–frequency domain by short-time Fourier transform was presented. The proposed method allowed detailed observation of the relationship between magnetic Barkhausen noise properties expressed in time and frequency for subsequent time moments [8].

The second group of papers has been devoted to the condition assessment of concrete and cement-based materials and structures [9,10,11,12,13,14,15,16,17]. Szeląg [9] presented a paper in which he reviewed the achievements in the field of the diagnostics of the cracking in cement composites. The four most important aspects related to the evaluation of a cracking pattern were addressed, namely: (i) the process of crack formation and their evolution into a branched system of cracks; (ii) the diagnostic techniques of digital extraction of the cracking patterns based on image analysis; (iii) the quantitative parameters used to describe the development degree and morphology of the cracks system; (iv) the influence of a cracking pattern on selected properties of cement composites. From the presented current state of the art in the field of the analysis of the cracking patterns in cement composites, it can be concluded that current works carried out in the field of crack assessment significantly contribute to the development of non-destructive testing methods in the field of cement composite technology [9]. The paper by Kuryłowicz-Cudowska [10] deals with the concept of a practical computation method to simulate the temperature distribution in a concrete structure. A model for the hardening of concrete, consistent with in situ measurement capabilities, was developed. Investigations consisted of three complementary parts: laboratory tests of high-performance concrete, measurements of temperature evolution in the bridge deck, and numerical simulations of temperature field in a concrete box bridge girder using the finite difference method. A new approach for the identification of the model parameters and heat transfer coefficients has been proposed. The results showed that the temperature history of concrete hardening, supplemented with maturity method equations, made it possible to estimate an early-age compressive strength of the cast-in-place concrete. It was also revealed that the proposed solution has great potential in the monitoring systems for concrete objects [10]. Słoński and Tekieli [11] presented a study on the monitoring and evaluation of surface cracks in concrete structural elements. The main goal was to integrate two computer vision methods, i.e., the digital image correlation (DIC) technique and region-based convolutional neural network (CNN) for the automatic detection and localization of multiple cracks. The proposed methodology was verified experimentally on post-tensioned, precast crane runway beams after more than 50 years of exploitation, subjected to three-point bending tests. At first, the DIC method was used for monitoring the deformation fields of the beam side surface. Next, the trained convolutional neural network was applied for the detection and localization of cracks. The results showed that the developed computer vision system was highly effective in automatically identifying the number of cracks and precisely pointing to their localization [11]. Sadowski et al. [12] proposed a complex methodology for the effective control of the entire process of executing floors made of cement-based materials. The methodology, developed based on many years of the authors’ experience, has three control stages: (i) control before starting the floor; (ii) ongoing control during the execution of the floor; (iii) control tests after finishing the execution of the floor. The authors indicated the need to use necessary semi-destructive and non-destructive methods for the examination of the technical condition of floors in various construction objects [12]. Perkowski and Tatara [13] investigated the detection of brittle damage in concrete beams using transmission ultrasonic tomography. Tomographic experiments were carried out on three reinforced concrete beams on a laboratory scale and one prefabricated beam on a natural scale. A method of reducing errors in the tomographic reconstruction of longitudinal wave velocity maps caused by the simplifying assumptions of the straightness of the fastest wave propagation paths was developed. The method consisted of the appropriate extension of the measured propagation times of the wave transmitted between opposite sending–receiving transducers if the actual propagation paths deviate from straight lines. The conducted research has shown that the accuracy of the tomography imaging of brittle damage in concrete could be effectively supported by the graph theory and Dijkstra’s algorithm [13]. Logoń and Schabowicz [14] used the acoustic emission (AE) method to recognize micro-events in quasi-brittle cement composites and to identify the destruction process. Experimental tests were conducted on a quasi-brittle composite of a cement paste reinforced with a high volume of dispersed polypropylene fibers. The main innovation of this research was the detection of AE micro-events using the sound spectrum in the area preceding the occurrence of critical cracks that initiate the destruction process in cement composites. It was confirmed that the application of the 3D spectrum enabled a better recognition of micro- and macro-changes in the structure of the samples based on the analysis of sound intensity, amplitudes, and frequencies [14]. Jasiński [15] evaluated changes in the stress state of masonry specimens made of autoclaved aerated concrete using the acoustoelastic method. The experimental studies were divided into two parts. At first, the empirical relationships between compressive stress and the velocity of the longitudinal ultrasonic wave, including humidity, were determined. Next, nine masonry walls were tested in axial compression. The obtained results showed that precise values of mean stress in the wall were determined on the basis of the measured velocity of ultrasonic wave propagation at a high number of measuring points, and the reduced number of measuring points resulted in a significant underestimation of mean stress [15]. The next two articles concerned bonding between a concrete substrate and cladding. Topolář et al. [16] experimentally examined the influence of the bonding system between a concrete substrate and large-format tiles. The bond strength was observed during mechanical loading under conditions that corresponded to real-life applications. Non-destructive diagnostics were performed using the following techniques: ultrasonic pulse velocity test, acoustic emission method, strain measurement, and acoustic tracing. The experiment showed that the loading caused no damage to the ceramic tile and all the detected failures took place in the adhesive layer or in the concrete slab [16]. Wojtczak et al. [17] applied the guided wave propagation technique for the condition assessment of concrete beams strengthened with steel plates. The novelty was in the comprehensive theoretical–numerical–experimental analysis of wave propagation in steel–concrete adhesively bonded specimens and the possibility of debonding imaging using weighted root mean square-based ultrasonic diagnostics. The research aimed at the damage identification and imaging in adhesive joints of composite beams with different levels of debonding. The experimental wave field was measured by scanning laser Doppler vibrometry. Next, the wave signals were processed by the weighted root mean square calculations. The obtained results indicated that the quality of damage maps strongly depended on the location of excitation [17].

Another group of articles concerned non-destructive testing of bridge structures [18,19,20]. Kwiatkowski et al. [18] presented a comparison of various measuring methods applied to investigate the behavior of a suspension bridge under different load scenarios. Three techniques, namely terrestrial laser scanning, tachymetry and photogrammetry, were examined on the bridge with a 165 m span. The testing range consisted of conducting the non-contact measuring of the bridge and cable displacements under dynamic and static loads and static loads. The obtained results enabled an assessment of the usefulness of the used measuring techniques. Some particular conclusions about the integration of the used methods have been drawn, especially in the context of measurements of structures under static and dynamic loading [18]. Mac et al. [19] focused on the detection of delamination in a concrete bridge deck. The main goal of the study was to develop a comprehensive system for bridge inspection using passive infrared thermography. Experimental tests were conducted on laboratory-scale concrete specimens with embedded artificial delamination of different width-to-depth ratios. Infrared cameras were employed to capture the surface temperature of the structure. The relations between the delamination size and depth and the heat energy were described. The optimal time to inspect the concrete bridge deck was determined [19]. Miśkiewicz et al. [20] described interdisciplinary and comprehensive non-destructive diagnostic tests of a soil–steel bridge made of corrugated sheets. A non-standard program of bridge loading was used during the inspection, including static and moving loads. The load test design was based on the finite element method simulations. In situ measurements were done by means of inductive sensors, an optical total station, and terrestrial laser scanner. The results obtained by terrestrial laser scanning were used to build a precise image of structural deformation. The accuracy of laser mapping was significantly increased using the information coming from the total station and the inductive sensors. A combination of testing methods was revealed as an effective tool in the non-destructive diagnostics of structures and an interesting alternative for the standard approach, in which the measurements are done in a limited number of points [20].

The diagnostics of cultural heritage structures were the subjects of interest in the last two papers from this Special Issue [21,22]. Tomaszewska et al. [21] investigated the problem of the modal identification of a masonry lighthouse. In the experiment, operational modal analysis was applied to the structure. Three identification techniques (peak picking, eigensystem realization algorithm and natural excitation technique with eigensystem realization algorithm) were applied to obtain modal parameters. The exact structural model was built by means of the finite element method. In the diagnostic process, the material properties were determined via numerical model validation applied to the first pair of natural frequencies and their related mode shapes, determined experimentally. As a result, the elastic modulus, Poisson’s ratio and material density of brick, sandstone and granite masonry were determined [21]. Rucka et al. [22] presented the results of the integrated ground penetrating radar (GPR) and ultrasonic testing (UT) inspection conducted on a historical floor. The aim of the study was to present the practical aspects of the application of both techniques in detecting and imaging the underfloor inclusions, such as air voids, brick walls, pipes, rubble and human remains. In order to better understand the phenomena of electromagnetic and ultrasonic wave propagation within the air voids and concentrated inclusions, laboratory tests were conducted on two concrete slabs stacked on top of each other and gradually moved apart to simulate a slot of varying thickness. The numerical simulations of electromagnetic waves were performed to support the interpretation of the GPR results. The obtained results showed that the integration of the GPR and UT methods provided effective imaging of the floor and the area under it. Ultrasonic testing proved to be a good technique for identifying air voids, while the GPR method allowed for the detection of concentrated anomalies and determining the degree of ground homogeneity under a floor. The presented research revealed the possibilities and limitations of both methods, indicating their complementarity in the context of the non-destructive diagnostics of historical buildings [22].

In summary, the papers presented in this Special Issue concern various aspects of non-invasive diagnostics and present recent developments in the field of the diagnostics of structural materials and components in civil and mechanical engineering. Both interesting practical solutions and a significant contribution to the new approaches to NDT were achieved.
